# Topical administration of interleukin-1 receptor antagonist as a therapy for aqueous-deficient dry eye in autoimmune disease

**Published:** 2013-09-19

**Authors:** Trinka Vijmasi, Feeling YT Chen, Ying Ting Chen, Marianne Gallup, Nancy McNamara

**Affiliations:** 1 Francis I. Proctor Foundation, University of California, San Francisco, 513 Parnassus Avenue, Rm. 1349, San Francisco, CA; 2 Department of Anatomy, University of California, San Francisco, San Francisco, CA; 3 Department of Ophthalmology, University of California at San Francisco, San Francisco, CA; 4 School of Optometry, University of California, Berkeley, Berkeley, CA

## Abstract

**Purpose:**

Dry eye is commonly associated with autoimmune diseases such as Sjögren’s syndrome (SS), in which exocrinopathy of the lacrimal gland leads to aqueous tear deficiency and keratoconjunctivitis sicca (KCS). KCS is among the most common and debilitating clinical manifestations of SS that is often recalcitrant to therapy. We established mice deficient in the autoimmune regulator (*Aire*) gene as a model for autoimmune-mediated aqueous-deficient dry eye. In *Aire*-deficient mice, CD4+ T cells represent the main effector cells and local signaling via the interleukin-1 (IL-1/IL-1R1) pathway provides an essential link between autoreactive CD4+ T cells and ocular surface disease. In the current study, we evaluated the efficacy of topical administration of IL-1R1 antagonist (IL-1RA) anakinra in alleviating ocular surface damage resulting from aqueous-deficient dry eye in the setting of autoimmune disease.

**Methods:**

We compared the effect of commercially available IL-1R1 antagonist, anakinra (50 μg/mL concentration) to that of carboxymethylcellulose (CMC) vehicle control as a treatment for dry eye. Age-matched, *Aire*-deficient mice were treated three times daily with anakinra or CMC vehicle for 14 days using side-by-side (n=4 mice/group) and paired-eye (n=5) comparisons. We assessed (1) ocular surface damage with lissamine green staining; (2) tear secretion with wetting of phenol-red threads; (3) goblet cell (GC) mucin glycosylation with lectin histochemistry; (4) immune cell infiltration using anti-F4/80, CD11c, and CD4 T cell antibodies; and (5) gene expression of cornified envelope protein, Small Proline-Rich Protein-1B (SPRR1B) with real-time quantitative polymerase chain reaction.

**Results:**

*Aire*-deficient mice treated with anakinra experienced significant improvements in ocular surface integrity and tear secretion. After 7 days of treatment, lissamine green staining decreased in eyes treated with anakinra compared to an equivalent increase in staining following treatment with CMC vehicle alone. By day 14, lissamine green staining in anakinra-treated eyes remained stable while eyes treated with CMC vehicle continued to worsen. Accordingly, there was a progressive decline in tear secretion in eyes treated with the CMC vehicle compared to a progressive increase in the anakinra-treated eyes over the 2-week treatment period. Aberrant acidification of GC mucins and pathological keratinization of the ocular surface were significantly reduced in anakinra-treated eyes. Significantly fewer Maackia amurensis leukoagglutinin positive goblet cells were noted in the conjunctiva of anakinra-treated eyes with a corresponding decrease in the expression of the pathological keratinization marker, SPRR1B. Finally, there was a downward trend in the infiltration of each immune cell type following anakinra treatment, but the cell counts compared to eyes treated with the vehicle alone were not significantly different.

**Conclusions:**

IL-1R antagonist, anakinra, demonstrates therapeutic benefits as a topical treatment for aqueous-deficient dry eye in a spontaneous mouse model of autoimmune KCS that mimics the clinical characteristics of SS. Targeting the IL-1/IL-1R1 signaling pathway through topical administration of IL-1RA may provide a novel option to improve ocular surface integrity, increase tear secretion, and restore the normal glycosylation pattern of GC mucins in patients with SS.

## Introduction

Dry eye disease (DED) is a common, debilitating disorder of the eye, affecting more than 25% of all people in the United States. In a comprehensive report, the dry eye workshop (DEWS) committee firmly established an essential role for inflammation in the pathogenesis of dry eye [1]. In autoimmune-mediated diseases like Sjögren’s syndrome (SS), exocrinopathy of the lacrimal gland leads to aqueous-deficient dry eye, known clinically as keratoconjunctivitis sicca (KCS). KCS is among the most common and debilitating clinical manifestations of SS, and as it progresses, the eye develops pathological characteristics that include (i) transdifferentiation of the ocular surface from a nonkeratinized, mucus-secreting epithelium to one that is pathologically keratinized and “skin-like” and (ii) altered glycosylation and loss of goblet cells (GCs). This process, known as squamous metaplasia (SQM), is a devastating, end-stage consequence of dry eye disease that can cause considerable morbidity as advanced keratinization couples with subepithelial fibrosis to cause corneal opacification and blindness. Although immune-mediated inflammation has been implicated in the pathogenesis of KCS and the development of SQM, little is known about the precise immunopathogenic mechanisms, and there is no cure.

To study the immunopathogenesis of autoimmune KCS in vivo, we and others have characterized a novel murine model of autoimmune-mediated dacryoadenitis with aqueous tear deficiency. Autoimmune REgulator gene knockout (*Aire* KO) mice spontaneously develop CD4+ T cell-mediated autoimmune disease that targets multiple organs, including the exocrine glands [2,3]. Accordingly, the exocrinopathy and ocular disease of *Aire* KO mice closely mimic the histopathological and clinical characteristics of SS. Through time course studies, we observed severe KCS, pathological keratinization, altered glycosylation of ocular surface O-glycans, loss of conjunctival GCs, and ocular infiltration of macrophages, major histocompatibility complex (MHC) class II+ antigen presenting cells (APCs), and CD4+ T cells. The onset of KCS largely depended on the presence of infiltrating immune cells, and through adoptive transfer studies, we identified autoantigen-primed CD4+ T cells as the primary effectors in the pathogenesis of autoimmune dry eye [4].

Although the specific proinflammatory mediators that provoke KCS are largely unknown, our laboratory and others have shown interleukin-1 (IL-1) cytokines are consistently and significantly elevated in the tears of patients with SS and the ocular surface expression of IL-1 cytokines was a significant predictor of epithelial cell damage [5,6]. Recently, we created a genetic knockout of IL-1 receptor in *Aire*-deficient mice and used adoptive transfer models to confirm the essential role of IL-1 signaling for initiating and perpetuating autoimmune KCS. Our studies were the first to establish local signaling via the IL-1/IL-1R1 pathway as a portal for KCS/SQM development in the setting of autoimmune disease [2].

With activation of IL-1 signaling an essential step in the pathogenesis of autoimmune KCS, the next step was to test whether local inhibition of the IL-1R1 pathway provided an effective therapy for aqueous-deficient dry eye. Although topical application of IL-1R1 antagonist is an effective therapy in mouse models of corneal injury and desiccating stress [7,8], the therapeutic potential for treating aqueous tear deficiency in autoimmune disease remains unknown. In this study, we report the therapeutic benefits of the U.S. Food and Drug Administration (FDA)-approved interleukin-1 receptor antagonist anakinra as a topical therapy for aqueous-deficient dry eye in a spontaneous mouse model of autoimmune KCS and Sjogren’s syndrome-like DED.

## Methods

### Materials

Lectins Maackia amurensis leukoagglutinin (MAL-1) and Sambucus nigra agglutinin (SNA) were purchased from Vector Labs (Burlingame, CA). Primary antibodies, rat antimouse CD4 and hamster antimouse CD11c, were from BD Pharmigen (San Jose, CA), and rat antimouse F4/80 was from AbD Serotec (Raleigh, NC). Secondary antibodies, Cy3-conjugated goat anti-Armenian hamster, Cy3-conjugated antirat, and horseradish peroxide–conjugated donkey antirat immunoglobulin G were from Jackson Laboratory (Bar Harbor, ME). The diaminobenzidine kit was from Dako North America (Carpentaria, CA). Antigen Retrieval Citrate Buffer (pH 6.0) was from Invitrogen (Carlsbad, CA). Fluorsav mounting medium was from Calbiochem (San Diego, CA). Pilocarpine was purchased from Sigma Aldrich (St. Louis, MO). Lissamine Green stain was from Leiter’s Pharmacy and Compounding Center (San Jose, CA). ZoneQuick Tear threads were from Showa Yakuhin Kako (Tokyo, Japan) distributed by Menicon American (San Mateo, CA). The High Capacity cDNA Reverse Transcription Kit and the TaqMan Universal Master PCR Mix for quantitative polymerase chain reaction (qPCR) were from Invitrogen. The primers for qPCR were from Applied Biosystems (Carlsbad, CA). Refresh Liquigel was developed by Allergan (Irvine, CA) while anakinra was developed by Amgen (Thousand Oaks, CA) and marketed as Kineret through Orphan Biovitrium (Sweden).

### Animals

Non-obese diabetic (NOD) mice with targeted mutation in the *Aire* gene were kind gifts from Dr. Mark Anderson, University of California, and San Francisco. Mice were housed in a pathogen-free barrier facility at UCSF. Offspring were genotyped for the *Aire* mutation with PCR of genomic DNA as previously described [2]. All experimental procedures adhered to animal care guidelines published in the U.S. Public Health Service Policy on Humane Care and Use of Laboratory Animals.

### Treatment regimen

*Aire* knockout (KO) mice on the NOD background were randomly allotted to either the control group or the experimental group at 5–6 weeks old. In this side-by-side comparison (n=4 mice/group), the control group received topical ocular administration of 50 μl of Refresh Liquigel lubricant (carboxymethylcellulose [CMC] vehicle), and the experimental group received 50 μl of anakinra (50 mg/ml, diluted in CMC), three times daily for 14 days. In a separate cohort of 5- to 6-week-old *Aire* KO mice, CMC vehicle and anakinra were assessed with paired-eye comparison (n=5) using an identical treatment regimen. Lissamine green staining and tear secretion assays were performed on both groups at days 0, 7, and 14.

### Lissamine green staining and quantification

Mice were anesthetized in an induction chamber filled with 3% isoflurane gas for 3-4 min and 5 µl of lissamine green dye (1%) was applied to the ocular surface. Photographs of the eye were taken using a Nikon Digital camera fitted to an Olympus Zoom Stereo Microscope (Olympus, Center Valley, PA). The images were scored on a 4-point scale by four independent, trained, masked observers. A score of 1 represents less than 25% of the ocular surface stained positive with lissamine dye, a score of 2 represents 25%–50% of the ocular surface stained positive, 3 represents 50%–75% of the ocular surface stained positive, and 4 represented greater than 75% of the ocular surface with positive staining. The average score from all four observers was used for each eye. The difference in the staining score at day 7 and day 14 relative to the baseline at day 0 is reported.

### Tear secretion assay

Tear secretion was quantified following intraperitoneal injection of 4.5 mg/kg pilocarpine. Ten minutes after injection, mice were anesthetized with isoflurane and secretion measured by the length of the tear-absorbed, color-changed region on a Zone-Quick phenol red thread. The difference in tear secretion, as measured by the mm of wetting of the phenol red thread, on day 7 and day 14 relative to the baseline at day 0 is reported.

### Immunohistochemical staining

Immune cells expressing F4/80, CD4, or CD11c were detected with immunofluorescence on frozen tissue sections fixed in acetone followed by washing with PBS-Tween (0.1%). After blocking for 1 h in serum derived from the species in which the secondary antibody was made, sections were incubated with primary antibodies at the following dilutions: F4/80 1:25, CD4 1:50, and CD11c 1:50 at 4 °C overnight. After washing with PBS-Tween, appropriate secondary antibody was added at a dilution of 1:200. For immunofluorescence staining, the nuclei were counterstained with 4',6-diamidino-2-phenylindole dihydrochloride and coverslipped with aqueous fluorescent preserving mounting medium, Fluorosav. For CD4 staining, the sections were incubated with diaminobenzidine, and the chromogen was developed per manufacturer's instructions. After rinsing in PBS, the sections were dehydrated and coverslipped in non-aqueous mounting medium.

### Lectin-histochemical staining

To determine the glycosylation pattern of mucins in the ocular surface, specifically the sialylation of mucins, lectins MAL-1 and SNA that are specific to sialic acid were used. Lectin staining involved deparafinizing the sections, followed by an antigen retrieval step in which the slides were boiled in the antigen retrieval citrate buffer (pH 6.0) at about 100 °C for 15 min in a water bath followed by 30 min of cooling in room temperature. After rinsing in PBS, the sections were blocked for 1 h in 3% bovine serum albumin and incubated with the biotinylated lectins MAL-I at 1:200 and SNA at 1:500 dilutions in antibody diluent (Dako) at 4 °C overnight. After the primary lectin was washed, the sections were incubated with Rhodamine conjugated Avidin at 1:500 for 1 h at room temperature. The sections were counterstained with 4',6-diamidino-2-phenylindole dihydrochloride and coverslipped with Fluorosav.

### Real-time quantitative polymerase chain reaction gene expression analysis

Real-time qPCR gene expression analysis was performed on corneolimbal sheets isolated from enucleated eyes, with a modification of our previously described protocol. Briefly, intact corneolimbal sheets were surgically peeled away from the underlying stroma of enucleated eyes following overnight enzymatic digestion with Dispase II (10 mg/ml) diluted in serum-free keratinocyte medium (Gibco) at 4 °C. Total RNA was extracted using RNeasy Mini RNA isolation kit (Qiagen, Hilden, Germany). Total RNA was eluted from minicolumns with 30 µl of RNase-free water. Starting from 1 µg of total RNA, 20 µl of cDNA was synthesized using the High Capacity cDNA Reverse Transcription Kit. The thermal cycling setup was according to the manufacturer’s instructions. Gene expression of Small Proline-Rich Protein-1B (SPRR1B) was measured in the ABI Prism 7300 Real-Time PCR System (Applied Biosystems) using the TaqMan Universal Master PCR mix kit as instructed by the manufacturer. All assays were performed in triplicate and normalized with the housekeeping gene, glyceraldehyde-3-phosphate dehydrogenase. The C_t_ data were derived from the untreated and treated eyes of four *Aire* KO mice and four wild-type (WT) mice.

### Statistical analysis

The Shapiro-Wilk W test was used to test for normal data. We used a Student’s *t* test or one-way analysis of variance (ANOVA) to examine statistical significance for normally distributed data, and the Kruskal–Wallis test for non-parametric analysis. The specific p values for each test are reported.

## Results

### Topical ocular therapy with anakinra restores ocular surface integrity and improves tear secretion

We tested the therapeutic potential of topical IL-1R1 antagonist anakinra as a treatment for aqueous-deficient dry eye in a spontaneous mouse model of autoimmune dry eye. *Aire*-deficient mice develop clinical features consistent with the human disease Sjögren’s syndrome with loss of epithelial integrity indicated by lissamine green staining and reduced tear secretion quantified with the phenol-red thread test.

**Figure 1:**
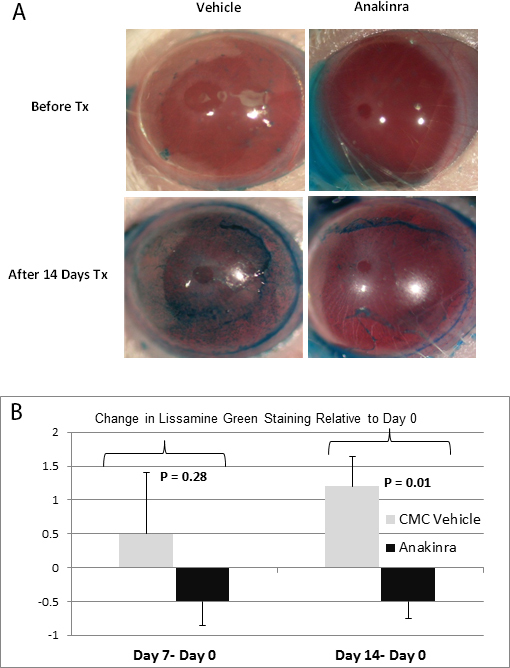
Anakinra improved ocular surface integrity. A 14-day course of topical anakinra significantly decreased progression of lissamine green staining in mice with autoimmune-mediated aqueous tear deficiency. **A**: Representative images of lissamine green staining for an autoimmune regulator (*Aire*) knockout (KO) mouse before (upper panels) and after (lower panels) 14-day treatment (Tx) with anakinra or carboxymethylcellulose (CMC) vehicle control. Staining was significantly reduced in anakinra-treated eyes (lower right) compared to those treated with vehicle (lower left). **B**: Lissamine green (LG) staining scores at days 7 and 14 are reported as a delta change relative to staining scores at baseline (day 0). The change in LG staining following treatment at day 7 (day 7– day 0) was 0.5±0.90 (CMC vehicle) versus −0.5±0.35 (anakinra); p=0.28, where positive values represent increased LG staining and negative values indicate decreased staining. By day 14 (day 14 – day 0), the change in LG reached 1.2±0.43 (CMC vehicle) versus −0.5±0.25 (anakinra); p=0.01).

In Figure 1, we report the change in lissamine green (LG) staining score relative to day 0, where a change in the positive direction represents an increase, or worsening, in LG staining. After 7 days of treatment, the mean ± standard error of the mean (SEM) levels of LG staining decreased in eyes treated with anakinra compared to an equivalent increase in staining following treatment with CMC vehicle alone (−0.5±0.35 [anakinra] versus 0.5±0.90 [CMC vehicle], p=0.28). By day 14, decreased levels of lissamine green staining in the anakinra-treated eyes remained stable while increased staining in eyes treated with CMC vehicle continued to progress (−0.5±0.25 [anakinra] versus 1.2±0.43 [CMC vehicle], p=0.01), leading to a significant difference between the two treatment conditions.

**Figure 2:**
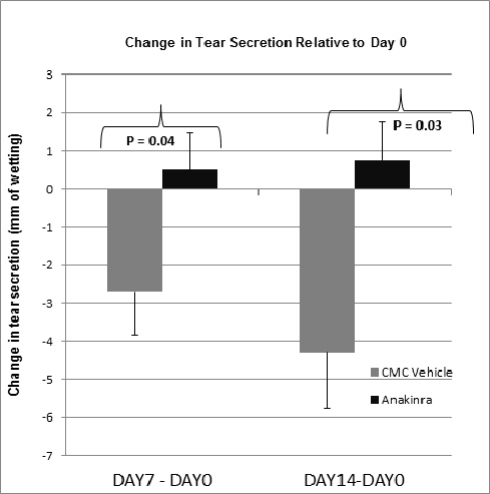
Anakinra improved tear secretion. A 14-day course of topical anakinra significantly increased tear secretion in mice with autoimmune-mediated aqueous tear deficiency. Changes in tear secretion at days 7 and 14 are reported relative to baseline secretion at day 0. Results are reported as millimeters of wetting measured using phenol-red threads. At day 7 (day 7 – day 0) changes in tear secretion were −2.7±1.1 (carboxymethylcellulose [CMC] vehicle) versus 0.49±0.20 (anakinra), p=0.04; where positive values represent increased tear secretion and negative values indicate decreased secretion. By day 14 (day 14 – day 0), changes in secretion were −4.3±1.4 (CMC vehicle) versus 0.74±0.0.49 (anakinra); p=0.03. Mice treated with CMC vehicle experienced a progressive decrease in tear secretion, whereas tear secretion increased in mice treated with anakinra and continued to improve throughout the 14-day protocol.

Accordingly, the amount of reflex tears secreted by eyes treated with anakinra significantly improved compared to eyes treated with the vehicle alone (Figure 2). Tear secretion in *Aire*-deficient mice, measured as millimeters of wetting using Quick-Zone Phenol red threads, gradually declined over time until reaching non-recordable levels around 8–9 weeks of age. Here, we report the change in tear secretion after 7 and 14 days of treatment relative to day 0 where a negative change indicates a decline in tear secretion and a positive change indicates improved secretion. On day 7, tear secretion declined in eyes treated with the CMC vehicle compared to a slight improvement in tear secretion for anakinra-treated eyes (0.49±0.20 [anakinra] versus −2.7±1.1 [CMC vehicle], p=0.04). By day 14, tear secretion continued to decline in eyes treated with the CMC vehicle and continued to improve in the anakinra-treated eyes (0.74±0.0.49 [anakinra] versus −4.3±1.4 [CMC vehicle], p=0.03).

### Restoration of goblet cell mucin glycosylation pattern

**Figure 3:**
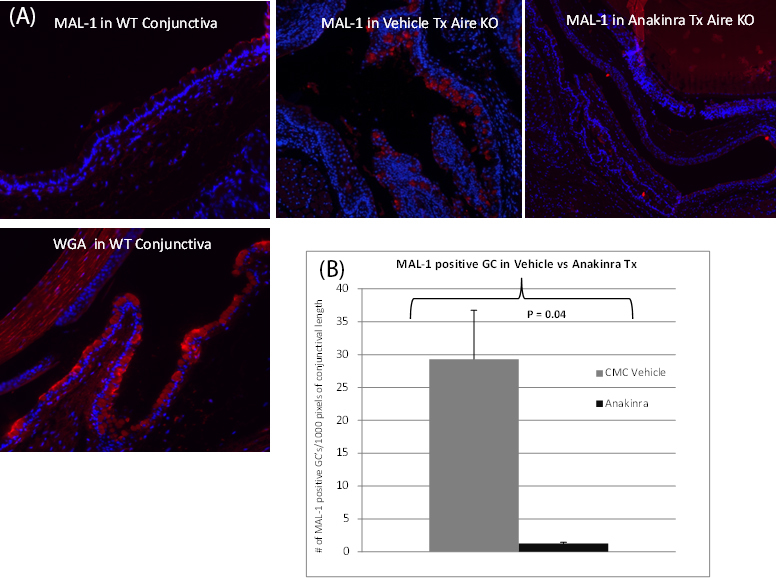
Anakinra restores normal goblet cell mucin glycosylation. Anakinra treatment significantly decreased the number of goblet cells (GCs) containing sialylated mucins in mice with autoimmune-mediated aqueous tear deficiency. **A**: Sialylated mucins were labeled using Maackia amurensis leukoagglutinin-1 (MAL-1) lectin. MAL-1-positive GCs were counted throughout the conjunctival epithelium of autoimmune regulator (*Aire*) knockout (KO) mice treated with anakinra or carboxymethylcellulose (CMC) vehicle control. Wheat germ agglutinin (WGA) staining of the conjunctiva used as positive control for goblet cell mucins. **B**: The total number of MAL-1+ GCs was 29.3±7.4 versus 1.26±0.18; p<0.05, for CMC vehicle versus anakinra-treated eyes, respectively, suggesting a reversal in aberrant GC mucin sialylation following inhibition of interleukin (IL)-1 signaling.

We assessed the conformation and extent of GC mucin sialylation with histochemistry using lectins with carbohydrate specificity for sialic acid-(α2,3)-Gal (β1,4; MAL-1) and sialic acid-(α2–6; SNA; Figure 3). There were no SNA-positive goblet cells in the conjunctiva. The number of goblet cells positive for MAL-1 were manually counted along the length of the conjunctiva and expressed as numbers per 1,000 pixel units of the length of the conjunctiva. We observed that the eyes treated with CMC vehicle had a significantly higher number of MAL-1-positive goblet cells in their conjunctiva compared to the anakinra-treated eyes (1.26±0.18 [anakinra] versus 29.3±7.4 [CMC vehicle]; p=0.04).

### Decreased gene expression of keratinization marker

**Figure 4:**
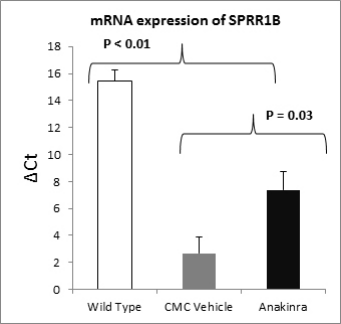
Anakinra reduced the expression of keratinization marker. Transcriptional profiling using real-time quantitative polymerase chain reaction (RT-qPCR) of messenger ribonucleic acid (mRNA) from corneolimbal sheets derived from the ocular surface of autoimmune regulator (*Aire*) knockout (KO) mice revealed that anakinra treatment significantly decreased ocular surface expression of keratin envelope protein, Small Proline-Rich Protein-1B (SPRR1B) (carboxymethylcellulose [CMC] vehicle ΔC_t_: 2.68±1.2 versus anakinra ΔC_t_: 7.39±1.3; p=0.03). A lower ΔC_t_ indicates higher expression levels.

SPRR1B expression is a significant predictor of ocular surface staining and ocular surface expression of IL-1β in human patients with SS and *Aire* KO mice [9]. To assess the effects of anakinra on SQM, we examined SPRR1B transcript levels in corneolimbal epithelial cells isolated from the ocular surface of the *Aire* KO mice treated with anakinra or CMC vehicle (Figure 4). The ΔC_t_, defined as the difference in the number of amplification cycles to threshold for the target gene, SPRR1B relative to the housekeeping gene, glyceraldehyde-3-phosphate dehydrogenase was calculated. A smaller ΔC_t_ indicates higher expression levels. We observed that the eyes treated with CMC vehicle had significantly more expression of the keratinization marker SPRR1B than the anakinra-treated eyes (ΔC_t_: 7.39±1.3 [anakinra] versus 2.68±1.2 [CMC vehicle]; p=0.03).

### Infiltration of immune cells

The number and pattern of infiltrating immune cells across the ocular surface of eyes treated with the CMC vehicle and anakinra were examined with immunohistochemistry using antibodies directed against F4/80+ macrophages, CD4+ T cells, and CD11c+ dendritic cells. Cells were manually counted and quantified separately for the limbus and the cornea. Cell numbers are expressed per 100,000 squared pixel area of the limbus and 1,000 pixel units of length of the cornea. As shown in Table 1, there was a downward trend in each immune cell type following anakinra treatment, but the differences in cell counts compared to eyes treated with the vehicle alone were not significantly different.

**Table 1:** Infiltration of immune cells

**Immune cell type**	**Limbus**		**Corneal stroma**	
	**CMC vehicle**	**Anakinra**	**CMC vehicle**	**Anakinra**
**CD4+ T cells**	33.8±2.4	24.2±2.8	16.7±0.1	13.2±0.2
**F4/80+ Macrophages**	12.6±1.3	8.9±3.2	4.1±1.8	1.89±0.24
**CD11c+ Dendritic cells**	2.27±0.07	0.73±0.35	0	0

Immune cells infiltrating the limbus and cornea were counted in the eyes of Aire KO mice treated with CMC vehicle or Anakinra. Mean ± SEM of cell counts are provided. Although there was a trend toward decreased immune cell numbers in Anakinra-treated eyes, differences did not reach statistical significance as defined by a p value <0.05.

## Discussion

With an improved understanding of dry eye pathogenesis over the past decade, the use of topical anti-inflammatory agents such as corticosteroids and the immunosuppressant, cyclosporine A, have gained steady popularity. However, the clinical response to prolonged use of non-specific, broad-acting anti-inflammatories varies, and significant side effects can occur [10,11]. Thus, developing highly targeted drugs to interrupt the inflammatory events that induce and sustain DED, and are safe for long-term use, is important.

Studies conducted by our laboratory and others have shown a prominent, mechanistic, immunomodulatory role for local IL-1 signaling in the pathogenesis of aqueous-deficient dry eye in autoimmune diseases, such as SS [2,6-8]. IL-1 is a pleiotropic, proinflammatory cytokine that is abundantly produced by mucosal epithelial and resident immune cells on the ocular surface. In a mouse model of CD4+ T cell-induced aqueous tear deficiency, we showed that sustained IL-1R1 activation sets off an aberrant inflammatory response that was firmly linked to ocular surface damage characterized by epithelial hyperplasia, epidermal-like differentiation, and pathological keratinization. Through the genetic knockdown of IL-1R1 and adoptive transfer studies, we demonstrated a significant reversal of ocular surface phenotype in the absence of local IL-1 signaling [2,4]. These studies led us to explore the potential therapeutic benefits of blocking IL-1/IL-1R1 signaling through the topical ocular application of commercially available IL-1R1 antagonist, anakinra.

The beneficial effects of anakinra for aqueous-deficient dry eye in *Aire* KO mice were in agreement with those reported using a validated model of induced DE mice where mice are placed in a low-humidity environmental chamber and maximum ocular dryness obtained through the topical application of 1% atropine and subcutaneous injection of scopolamine [8]. The immunomodulatory role of IL-1 has been demonstrated in corneal wound healing, as well, where topical application of interneukin-1 receptor antagonist markedly reduced corneal infiltration of immune cells in a mouse model of injury-induced inflammation [7]. In a recent randomized clinical trial study involving 75 patients with dry eye disease, the therapeutic use of topical anakinra was clearly demonstrated [12]. These previous studies lend strong support to anakinra’s promise as a topical therapeutic for ocular surface inflammation, while our findings extend these therapeutic benefits to a model of ocular pathology associated with autoimmune disease. Notably, the *Aire* KO mouse differs from other models in that it spontaneously develops an aqueous-deficient dry eye that closely mimics the natural development of autoimmune ocular surface disease, such as that observed in Sjögren’s syndrome. Using this in vivo model, we have, for the first time, shown the potential therapeutic benefits of topical ophthalmic anakinra for treating less common, but more clinically recalcitrant, autoimmune-mediated aqueous deficient dry eye.

In addition to anakinra’s positive impact on mucosal integrity, we noted increased tear secretion within 1 week of initiating topical therapy with anakinra. Dry eye disease is widely considered an inflammatory disease of the lacrimal functional unit (LFU), defined as an integrated system comprised of the lacrimal glands and ocular surface, including the cornea, the conjunctiva, and the sensory and motor nerves that connect them [13]. The cornea is highly innervated, and growing evidence shows lacrimal tear secretion is tightly regulated by corneal innervation [14]. We have unpublished data demonstrating decreased corneal innervation in *Aire* KO mice. Thus, the repair of mucosal epithelial integrity in response to topical anakinra deserves further study to determine whether it enhances tear secretion by promoting the restoration of corneal sensory function.

In conjunction with anakinra’s promising effects on ocular surface integrity and tear secretion, we observed restoration of the natural glycosylation pattern of GC mucins. Mucins are an integral component of the tear film and essential for maintaining tear film stability. Modification of secreted mucins can provoke changes in lubrication and protection of the mucosal surface. Decreased and altered ocular mucin glycoconjugates have been reported in DED, as well as in autoimmune diseases such as ocular cicatricial pemphigoid, Stevens-Johnson syndrome, and Sjögren’s syndrome [15]. Robust acidification of GC mucins in *Aire* KO mice was reversed when *Aire*-deficient mice lacked functional IL-1R1, demonstrating the essential role of local IL-1 signaling in altered glycosylation [5]. Acidification of GC mucins can occur via the terminal addition of negatively charged sialic acid or sulfated-carbohydrate residues. Using histochemical stains and sialic acid-specific lectins, we discovered that the link between IL-1R1-mediated ocular inflammation and GC mucin acidification occurred as the result of increased sialylation of terminal galactose residues via galactosyl (α-2,3) bonds. Moreover, we identified topical anakinra as an effective therapy for restoring the natural glycosylation pattern of GC mucins in the setting of chronic inflammation.

Acidification of GC mucins during ocular surface inflammation is not unprecedented and has been reported in the tears of human patients with ocular rosacea [16]. Sialic acids are most often the terminal sugar in the glycoprotein. They are readily available candidates for recognition and signaling and, thus, perform important roles in immune regulation and inflammation [17,18]. Because sialic acids are abundantly expressed on several kinds of mammalian cells, the specific linkage carries great significance for immune recognition [19]. For instance, in the immune system, T-cells and macrophages widely express Siglec-1 a sialic-acid-binding immunoglobulin-like lectin that specifically binds α2, 3, α2, 6, and α2, 8-linked sialic acids expressed on multiple cells types, including mucosal epithelial cells [20]. Siglec-1-expressing macrophages have been shown to increase the proliferative capacity of CD4+ T cells and promote interferon gamma secretion [21,22]. In our own studies of *Aire* KO mice, we found an essential role for macrophage infiltration in the pathogenesis of CD4+ T cell-mediated dry eye disease where a high density of macrophage infiltration was observed in the GC-rich region of the conjunctiva of inflamed eyes [23]. Whether the aberrant α2, 3 sialylation of mucins observed in *Aire* KO mice contributed to the infiltration of macrophages is unknown, but additional studies on the role of specific sialic acid linkages may improve our understanding of the underlying mechanism.

Finally, we did not see significant differences in immune cell infiltration of the corneolimbal area of *Aire* KO mice following anakinra treatment. This result was in line with our previous studies, where knockdown of IL-1R1 signaling in *Aire* KO mice led to significant improvements in the ocular phenotype without notable changes in the CD11c+ dendritic cells or autoreactive CD4+ T cell infiltration. These observations support the notion that anakinra’s therapeutic effect occurs locally, downstream of resident and infiltrating immune cells. Thus, anakinra provides a powerful yet targeted therapy for treating dry eye where the prosecretory and mitogenic effects promote ocular surface health despite persistent T-cell infiltration.

We used *Aire*-deficient mice as a model of autoimmune-mediated aqueous-deficient dry eye that mimics the clinical characteristics of SS-KCS to demonstrate the therapeutic potential of the topically applied IL-1R1 antagonist anakinra. Anakinra improved ocular surface integrity, increased lacrimal gland tear secretion, restored the glycosylation pattern of conjunctival goblet cells mucins, and decreased pathological keratinization of the ocular surface. These data provide support for further testing this FDA-approved therapeutic as a standalone or adjunct therapy to treat inflammatory conditions of the ocular surface that occur in patients with autoimmune disease.

## References

[1] (2007). Research in dry eye: report of the Research Subcommittee of the International Dry Eye WorkShop (2007).. Ocul Surf.

[2] Chen YT, Nikulina K, Lazarev S, Bahrami AF, Noble LB, Gallup M, McNamara NA (2010). Interleukin-1 as a phenotypic immunomodulator in keratinizing squamous metaplasia of the ocular surface in Sjogren's syndrome.. Am J Pathol.

[3] Chen YT, Li S, Nikulina K, Porco T, Gallup M, McNamara N (2009). Immune profile of squamous metaplasia development in autoimmune regulator-deficient dry eye.. Mol Vis.

[4] Chen YT, Lazarev S, Bahrami AF, Noble LB, Chen FY, Zhou D, Gallup M, Yadav M, McNamara NA (2012). Interleukin-1 receptor mediates the interplay between CD4(+) T cells and ocular resident cells to promote keratinizing squamous metaplasia in Sjogren's syndrome.. Lab Invest.

[5] Li S, Gallup M, Chen YT, McNamara N (2010). Molecular Mechanism of Proinflammatory Cytokine-Mediated Squamous Metaplasia in Human Corneal Epithelial Cells.. Invest Ophthalmol Vis Sci.

[6] Solomon A, Dursun D, Liu Z, Xie Y, Macri A, Pflugfelder SC (2001). Pro- and anti-inflammatory forms of interleukin-1 in the tear fluid and conjunctiva of patients with dry-eye disease.. Invest Ophthalmol Vis Sci.

[7] Stapleton WM, Chaurasia SS, Medeiros FW, Mohan RR, Sinha S, Wilson SE (2008). Topical interleukin-1 receptor antagonist inhibits inflammatory cell infiltration into the cornea.. Exp Eye Res.

[8] Okanobo A, Chauhan SK, Dastjerdi MH, Kodati S, Dana R (2012). Efficacy of topical blockade of interleukin-1 in experimental dry eye disease.. Am J Ophthalmol.

[9] Li S, Nikulina K, DeVoss J, Wu AJ, Strauss EC, Anderson MS, McNamara NA (2008). Small proline-rich protein 1B (SPRR1B) is a biomarker for squamous metaplasia in dry eye disease.. Invest Ophthalmol Vis Sci.

[10] Pflugfelder SC (2004). Antiinflammatory therapy for dry eye.. Am J Ophthalmol.

[11] McCabe E, Narayanan S (2009). Advancements in anti-inflammatory therapy for dry eye syndrome.. Optometry.

[12] Amparo F, Dastjerdi MH, Okanobo A, Ferrari G, Smaga L, Hamrah P, Jurkunas U, Schaumberg DA, Dana R (2013). Topical interleukin 1 receptor antagonist for treatment of dry eye disease: a randomized clinical trial.. JAMA Ophthalmol.

[13] (2007). The definition and classification of dry eye disease: report of the Definition and Classification Subcommittee of the International Dry Eye WorkShop (2007).. Ocul Surf.

[14] Dartt DA (2009). Neural regulation of lacrimal gland secretory processes: relevance in dry eye diseases.. Prog Retin Eye Res.

[15] Gipson IK, Hori Y, Argueso P (2004). Character of ocular surface mucins and their alteration in dry eye disease.. Ocul Surf.

[16] An HJ, Ninonuevo M, Aguilan J, Liu H, Lebrilla CB, Alvarenga LS, Mannis MJ (2005). Glycomics analyses of tear fluid for the diagnostic detection of ocular rosacea.. J Proteome Res.

[17] Varki A, Gagneux P (2012). Multifarious roles of sialic acids in immunity.. Ann N Y Acad Sci.

[18] Schauer R (2009). Sialic acids as regulators of molecular and cellular interactions.. Curr Opin Struct Biol.

[19] Marth JD, Grewal PK (2008). Mammalian glycosylation in immunity.. Nat Rev Immunol.

[20] Crocker PR, Paulson JC, Varki A (2007). Siglecs and their roles in the immune system.. Nat Rev Immunol.

[21] Crocker PR, Varki A (2001). Siglecs, sialic acids and innate immunity.. Trends Immunol.

[22] Pillai S, Netravali IA, Cariappa A, Mattoo H (2012). Siglecs and immune regulation.. Annu Rev Immunol.

[23] Zhou D, Chen YT, Chen F, Gallup M, Vijmasi T, Bahrami AF, Noble LB, van Rooijen N, McNamara NA (2012). Critical involvement of macrophage infiltration in the development of Sjogren's syndrome-associated dry eye.. Am J Pathol.

